# Five-year disease-free survival among stage II-IV breast cancer patients receiving FAC and AC chemotherapy in phase II clinical trials of Panagen

**DOI:** 10.1186/s12885-016-2711-5

**Published:** 2016-08-18

**Authors:** Anastasia S. Proskurina, Tatiana S. Gvozdeva, Ekaterina A. Potter, Evgenia V. Dolgova, Konstantin E. Orishchenko, Valeriy P. Nikolin, Nelly A. Popova, Sergey V. Sidorov, Elena R. Chernykh, Alexandr A. Ostanin, Olga Y. Leplina, Victoria V. Dvornichenko, Dmitriy M. Ponomarenko, Galina S. Soldatova, Nikolay A. Varaksin, Tatiana G. Ryabicheva, Peter N. Uchakin, Vladimir A. Rogachev, Mikhail A. Shurdov, Sergey S. Bogachev

**Affiliations:** 1 Institute of Cytology and Genetics, Siberian Branch of the Russian Academy of Sciences, 10 Lavrentieva Ave, Novosibirsk, 630090 Russia; 2 Novosibirsk State Medical University, Novosibirsk, 630091 Russia; 3 Novosibirsk State University, Novosibirsk, 630090 Russia; 4 Oncology Department of Municipal Hospital No 1, Novosibirsk, 630047 Russia; 5 Institute of Clinical Immunology, Siberian Branch of the Russian Academy of Medical Sciences, Novosibirsk, 630099 Russia; 6 Irkutsk State Medical Academy of Postgraduate Education, Irkutsk, 664049 Russia; 7 Regional Oncology Dispensary, Irkutsk, 664035 Russia; 8 Clinic Department of the Central Clinical Hospital, Siberian Branch of the Russian Academy of Sciences, Novosibirsk, 630090 Russia; 9 CJSC “Vector-best”, Koltsovo, Novosibirsk Region 630559 Russia; 10 Mercer University School of Medicine, Macon, GA 31207 USA; 11 LLC “Panagen”, Gorno-Altaisk, 649000 Russia

**Keywords:** Breast cancer, FAC chemotherapy, AC chemotherapy, Disease-free survival, dsDNA, CD8 + perforin + T cells

## Abstract

**Background:**

We report on the results of a phase II clinical trial of Panagen (tablet form of fragmented human DNA preparation) in breast cancer patients (placebo group *n* = 23, Panagen *n* = 57). Panagen was administered as an adjuvant leukoprotective agent in FAC and AC chemotherapy regimens. Pre-clinical studies clearly indicate that Panagen acts by activating dendritic cells and induces the development of adaptive anticancer immune response.

**Methods:**

We analyzed 5-year disease-free survival of patients recruited into the trial.

**Results:**

Five-year disease-free survival in the placebo group was 40 % (*n* = 15), compared with the Panagen arm – 53 % (*n* = 51). Among stage III patients, disease-free survival was 25 and 52 % for placebo (*n* = 8) and Panagen (*n* = 25) groups, respectively. Disease-free survival of patients with IIIB + C stage was as follows: placebo (*n* = 6)–17 % vs Panagen (*n* = 18)–50 %.

**Conclusions:**

Disease-free survival rate (17 %) of patients with IIIB + C stage breast cancer receiving standard of care therapy is within the global range. Patients who additionally received Panagen demonstrate a significantly improved disease-free survival rate of 50 %. This confirms anticancer activity of Panagen.

**Trial registration:**

ClinicalTrials.gov NCT02115984 from 04/07/2014.

## Background

Breast cancer along with skin, lung, colorectal and stomach cancer make up the top five most common malignancies. It is a leading cause of cancer-related mortality among women and therefore has a high social impact [1–3]. Positive prognosis in breast cancer is correlated with early diagnosis and proper choice of systemic therapy. Presently, the conventional breast cancer therapies include mastectomy or radical resection, adjuvant chemotherapy, hormonal therapy, and targeted therapy (whenever appropriate, such as in HER2/neu-positive tumors) (reviewed in [4]). Given that breast cancer is a systemic disease, cytoreductive chemotherapy is essential for the successful treatment.

In breast cancer, chemotherapy combines alkylating activity of a cytostatic drug (cyclophosphamide), anthracycline (doxorubicin, farmorubicin, mitoxantrone) and antimetabolite (5-fluorouracil, ftorafur, gemcitabine, xeloda, metotrexate). Treatment schemes may also include Vinca alkaloids (vincristine, vinblastine, navelbine), taxanes (taxol, taxotere) and platinum compounds (cisplatin, carboplatin). Two basic schemes remain at the mainstream of breast cancer therapy: FAC (a combination of 5-fluorouracil, doxorubicin and cyclophosphamide) and AC (doxorubicin and cyclophosphamide). To boost the efficiency, these can be further modified by introducing additional components or substituting the basic components with other drugs [5–10].

As much as 1.5 million new cases of breast cancer are diagnosed worldwide each year, with a lethal outcome for about 400 thousand people. The current gold standard for assessing the therapeutic efficacy in breast cancer patients is 5-year disease-free survival (hereafter referred to as DFS), i.e. the percentage of patients that are alive 5 years after their primary treatment without any signs or symptoms of that cancer. Whereas stage II breast cancer is curable, with DFS ranging 75–90 %, and stage IV breast cancer has a poor prognosis with DFS of 0–10 %, efficacy of the therapy is best established by assessing DFS of stage III patients.

From the clinical perspective, locally disseminated breast cancer (stage III) has DFS rate of 10–50 % regardless of the frontline therapy received by the patient [4, 6, 11, 12]. Stage III breast cancer can be further subdivided into three subgroups IIIA, B and C, according to the extent of disease progression [6, 12]. Stages IIIB and IIIC consistently have a poorer prognosis compared to IIIA, which is mirrored by DFS in these patient groups below ~30 % [12].

Our previous report [13] summarizes the results of phase II clinical trial of Panagen with major emphasis on the analysis of Panagen’s leukoprotective properties. The data obtained in the study indicate that inclusion of Panagen into standard chemotherapy regimens leads to protection of white blood cell lineage from detrimental effects of three consecutive rounds of FAC or AC. Notably, combination of Panagen with either FAC or AC therapies results in fewer grade I-IV neutropenias and in the maintenance of pre-therapeutic activity of innate anticancer immunity cells.

It was first reported in 2001 that genomic double-stranded DNA as well as CpG oligodeoxynucleotides can activate antigen-presenting properties of dendritic cells and that it boosts the developing adaptive immune response [14]. In later studies, the major focus was on CpG DNA as a structurally homogeneous molecule with a therapeutic potential [15, 16]. In contrast, genomic double-stranded DNA received far less attention in this respect due to the issues in standardization of double-stranded DNA preparations. Our group, however, continued to explore and analyze how fragmented human double-stranded DNA influences different cell types and cell populations, using mouse models and in clinical trials.

Our earlier studies focused on unraveling how double-stranded DNA-based medications may interact with the body cellular machinery. These studies demonstrated that the primary cell targets of fragmented exogenous double-stranded DNA are peripheral blood mononuclear cells, dendritic cells, as well as stem cells of various origin [17–19]. It is this interaction of Panagen with peripheral blood mononuclear and dendritic cells that mediates its leukoprotective activity and the development of adaptive anticancer immunity [20, 21]. With this in mind, when performing stage II clinical trial, we designed and implemented an additional experimental protocol that was supposed to inform us more on the development of adaptive anticancer immune response in patients recruited to the study. We showed that in the patient group receiving Panagen, there is a statistically significant increase in the percentage of CD8 + perforin + cytotoxic T cells in peripheral blood, − importantly, it is this cell type that is known to play one of the major roles in adaptive immunity.

Early experiments showed pronounced activation of dendritic cells upon CpG DNA administration. This effect was mediated by the interaction of the ligand and TLR9 in dendritic cells [14–16]. Subsequently, several types of CpG oligodeoxynucleotides were tested as anticancer agents in the context of breast cancer [22, 23]. In contrast to our approach, wherein tablet form of Panagen was used per os and the active substance primarily targeted the mucosal immune cells, all the CpG-based medications were delivered as intravenous, intramuscular or subcutaneous injections. Data obtained in phase I and II clinical trials indicated that these medications were highly toxic to the trial participants and caused a number of serious side effects. For this reason, all the clinical trials of these drugs as anticancer agents are currently put on hold. Instead, the interest in these drugs has shifted to their possible use as adjuvants and to designing the CpG-based medications that are less toxic [22, 23]. Our phase II clinical trial of Panagen was based on the premise that activation of dendritic cells resident in the intestinal mucosa (using gastro-resistant tablets) should be comparable to the injection form of CpG-based preparations in terms of inducing adaptive immunity, yet it should have a favorable toxicity profile.

In the present report, we analyze the efficiency of Panagen as an adjuvant anticancer medication, given that it can enhance personalized anticancer adaptive immunity [13]. Specifically, we calculated and compared 5-year DFS of patients from experimental and placebo groups. This analysis also helped determine the breast cancer stage when combined use of Panagen and the standard chemotherapy (FAC or AC) results in the best response rate.

## Methods

Phase II clinical trial of preparation Panagen was approved by the Ministry of Health and Social Development of the Russian Federation (No. 47 of 03/12/2010) as well as by the local ethics committees at the Irkutsk Regional Oncology Dispensary and the Novosibirsk Municipal Hospital No 1, where clinical trials were subsequently performed. The studies were carried out in compliance with the World Medical Association Declaration of Helsinki. Written informed consent to participate in the study was obtained from each of the patients, which specified open publication of the results presented as reports or otherwise. All patients were also insured.

Other details of the clinical trial protocol can be found in [13].

## Results

### Overall analysis of 5-year survival of patients recruited to the phase II clinical trial of Panagen

Patients recruited to the clinical trials of Panagen were followed-up for 5 years after the therapy. Overall, of 80 stage II-IV breast cancer patients 13 were excluded from the study for various reasons. Thus, the Panagen and the placebo arms of the trial included 51 and 16 patients, respectively. All the patients completed the full course of FAC or AC therapies followed by tamoxifen therapy, whenever their tumors were classified as hormone-dependent. By the end of the 5-year period data of survival were collected (Table 1).

**Table 1:** Patient data during the clinical trial of Panagen

Patient number	Breast cancer stage		1 year	1.5 years	2 years	3–4 years	5 years
FAC chemotherapy + Placebo							
01–03	T2N1M0	IIB	remission	remission	remission	remission	remission
01–08	T2N0M0	IIA	remission	remission	remission	remission	remission
01–12	T4N3M1	IV	remission	remission	disease progression	deceased	–
01–16	T4N2M0	IIIB	remission	remission	deceased (cause of death is not cancer)	-	-
01–23	T4N0M0	IIIB	remission	remission	disease progression	no data	no data
02–07	T4N2M0	IIIB	deceased	-	-	-	-
02–12^a^	T2N2M0	IIIA	remission	remission	no data	no data	no data
02–13	T4N3M0/1	IV	therapy continues	disease progression, lung metastases	deceased	-	-
02–17	T2N1M0	IIB	remission	remission	remission	remission	remission
AC chemotherapy + Placebo							
02–23^a^	T2N1M0/1	IV	disease progression, lymph node metastases before the study + bone metastases	deceased	-	-	-
02–32	T4NxM0	IIIB	remission	remission	remission	remission	no data
02–34	T4N2M0	IIIB	disease progression, metastases to bones, lungs, and soft tissues of the chest wall	no data	no data	no data	no data
02–35^a^	T4NxM0	IIIB	remission	no data	no data	deceased	-
02–37^a^	T4N0M0	IIIB	disease progression, brain, lungs, liver and bone metastases	deceased	-	-	-
02–38	T2N2M0	IIIA	no data	deceased	-	-	-
02–41	T1N1M0	IIA	remission	remission	remission	remission	no data
FAC chemotherapy + Panagen							
01–01	T1N2M0	IIIA	remission	remission	remission	disease progression	no data
01–02	T1N2M0	IIIA	remission	remission	remission	disease progression	no data
01–04	T4N2M1	IV	remission	remission	remission	deceased	-
01–05	T2N1M0	IIB	remission	deceased	-	-	-
01–06	T4N1M0	IIIB	remission	remission	remission	remission	remission
01–07	T3N0M0	IIB	remission	remission	remission	remission	remission
01–09	T2N2M0	IIIA	remission	remission	remission	no data	no data
01–10	T4N3M0	IIIC	remission	remission	remission	remission	no data
01–11	T2N1M0	IIB	remission	remission	remission	remission	no data
01–13	T4N3M0	IIIC	remission	remission	remission	remission	remission
01–14	T4N2M0	IIIB	remission	remission	remission	disease progression	deceased
01–15	T2N2M0	IIIA	remission	remission	remission	remission	remission
01–17	T2N1M0	IIB	remission	remission	remission	no data	no data
01–18	T4N1M0	IIIB	disease progression	no data	no data	no data	alive
01–19	T4N1M0	IIIB	remission	remission	remission	deceased	–
01–20	T2N0M0	IIA	remission	remission	remission	remission	remission
01–21	T4N3M0	IIIC	disease progression	no data	no data	alive	alive
01–22	T4N1M1	IV	remission	remission	remission	disease progression	deceased
02–01	T4NxM0	IIIB	no data	no data	no data	disease progression, tumor disintegration, multiple metastases in lungs	deceased
02–02	T2N0M0	IIA	remission	remission	remission	no data	no data
02–03	T1N1M0	IIA	remission	remission	remission	disease progression, Th11 metastases	no data
02–04	T3N2M1	IV	deceased	-	-	-	-
02–05	T4N1M0	IIIB	remission	remission	remission	remission	remission
02–06	T4NxM1	IV	deceased	-	-	-	-
02–08	T4N1M0	IIIB	remission	remission	remission	disease progression, lung, pleura and liver metastases	no data
02–09	T1N1M0/1	IV	disease progression, bone metastases observed during the 1^st^ CT round	alive	alive	deceased	-
02–10	T1N1M0	IIA	remission	remission	remission	remission	remission
02–11^a^	T2N1M0	IIB	remission	remission	remission	remission	remission
02–14	T2N2M0dex T4N1M0sin	IIIB	remission	remission	remission	remission	remission
02–15	T3N2M0	IIIA	remission	remission	remission	remission	remission
02–16^a^	T4NxM0	IIIB	remission	disease progression, metastases in the skin region adjacent to surgery scar	partial regression	deceased	-
02–18^a^	T4NxM0	IIIB	remission	remission	remission	remission	remission
AC chemotherapy + Panagen							
02–20	T2N1M0	IIB	remission	remission	remission	disease progression, bone metastases	no data
02–21	T2N0M0	IIA	remission	remission	remission	remission	no data
02–22^a^	T2N1M0	IIB	remission	remission	remission	remission	no data
02–24	T2N1M0	IIB	remission	remission	remission	remission	no data
02–25^a^	T2N2M0	IIIA	remission	remission	remission	remission	no data
02–26	T1N1M0	IIA	remission	remission	remission	remission	no data
02–27	T1N2M0	IIIA	remission	remission	disease progression, bone metastases	no data	no data
02–28^a^	T2N1M0	IIB	remission	remission	disease progression, bone and lung metastases	no data	no data
02–29	T2N0M0	IIA	remission	remission	no data	deceased	-
02–30	T2NxM0	II	remission	remission	remission	remission	no data
02–31	T2N1M0/1	IV	disease progression, lung metastases observed 1 month following completion of the therapy	no data	no data	no data	no data
02–33^a^	T2N3M0	IIIC	remission	remission	remission	remission	no data
02–36	T4NxM0dex T2NxM0sin	IIIB	treatment continues	treatment continues	no data	no data	no data
02–39	T2N3M0	IIIC	remission	remission	relapse	no data	no data
02–40^a^	T2N1M0	IIB	remission	no data	no data	no data	no data
02–42^a^	T1N3M0	IIIC	remission	remission	remission	remission	no data
02–43	T3N0M0	IIB	remission	remission	remission	remission	no data
02–44^a^	T1N1M0/1	IV	disease progression, bone metastases observed after the 2^nd^CT round	no data	no data	no data	no data
02–45^a^	T4N1M0	IIIB	remission	remission	disease progression, pleural metastases	no data	no data

Note: ^a^ – tamoxifen treatment. *CT* chemotherapy. Patients who progressed or died are shown in boldface. Cause of death in all cases is breast cancer, except patient 01–16. Patient 01–16, whose cause of death was not cancer, didn’t taken into account further

We analyzed 5-year overall survival and DFS of patients recruited to the study. These parameters were calculated separately for disease stages. Additionally, we estimated the correlation between 5-year DFS and the immune status of FAC-treated patients.

### Five-year overall survival and DFS of patients in Panagen trial

Our analysis suggests that 5-year DFS of patients from the placebo group was 40 %, whereas for those receiving Panagen it was 53 % (Table 2).

**Table 2:** Five-year disease-free survival

	Chemotherapy + Placebo			Chemotherapy + Panagen			[38]
	Patients	Survived patients	%	Patients	Survived patients	%	Survival, %
I							76.7
II	4	4	100	19	14	74	
III	8	2	25	25	13	52	
IV	3	0	0	7	0	0	
Total	15	6	40	51	27	53	

Of 8 stage III placebo-group patients, 6 had IIIB or IIIC breast cancer. The same substages were diagnosed for 18 patients out of 25 stage III breast cancer patients in the Panagen-group. Five-year DFS of IIIB/IIIC patients in the placebo group was 17 %, compared to 50 % observed in the Panagen group (Table 3).

**Table 3:** Five-year disease-free survival for stage III breast cancer

	Chemotherapy + Placebo				Chemotherapy + Panagen				[25]
	Patients	Survived patients	%		Patients	Survived patients	%		Survival, %
IIIA	2	1		50	7	4		57	47
IIIB	6	1	17	17	12	5	42	50	
IIIC	0	-	-		6	4	67		
Stage III, total	8	2		25	25	13		52	

Next, survival of patients in our trial was compared to the literature data. Both 5-year DFS and overall survival are consistent with the current literature rates (Tables 2 and 4). Overall survival of substage IIIA and IIIB breast cancer patients in the placebo cohort was comparable to the figures referenced in the literature (Table 5). Notably, in the Panagen arm, overall survival of stage III patients was significantly higher than that in the literature. Namely, for substage IIIA patients the numbers were 100 % (Panagen) vs 66.7 % [24], for stage IIIB, overall survival was 67 % (Panagen) vs 41 % [24], and for IIIA and IIIB substages the combined overall survival was 79 % (Panagen) vs 57 % [25]. Importantly, overall survival of substage IIIC patients on Panagen was 100 %. This data of 5-year overall survival for stage III show a significant contribution of Panagen to the treatment efficiency. Differences in the 5-year DFS also support the contribution of Panagen to favorable outcome (Table 3).

**Table 4:** Five-year overall survival

	Chemotherapy + Placebo			Chemotherapy + Panagen			[38]	[24]	[39]
	Patients	Survived patients	%	Patients	Survived patients	%	Survival, %	Survival, %	Survival, %
I							100.0	92.1	
II	4	4	100	19	17	89	89.0	81.8	
III	8	4	50	25	21	84	80.8	58.0	
IV	3	0	0	7	2	29			
Total	15	8	53	51	40	78			40–70

**Table 5:** Five-year overall survival for stage III breast cancer

	Chemotherapy + Placebo				Chemotherapy + Panagen				[25]	[24]
	Patients	Survived patients	%		Patients	Survived patients	%		Survival, %	Survival, %
IIIA	2	1	50	50	7	7	100	79	57	66.7
IIIB	6	3	50		12	8	67			41
IIIC	0	-		-	6	6		100		
Stage III, total	8	4		50	25	21		84		

### Comparison of immune status of patients on FAC + Placebo vs FAC + Panagen regimens

Several parameters informative of the immune status of the patients were measured in the additional protocol of the clinical study. These include changes in cell counts for CD123+ (plasmacytoid dendritic cells), CD11+ (myeloid dendritic cells), CD25+ CD127– (T-regs), CD8 + perforin + (cytotoxic T-cells). Higher counts of CD123+, CD11+ and CD8 + perforin + cells in patients would be interpreted as activated adaptive immunity. Decreased T-reg counts are generally indicative of the reduced immunosuppression by the tumor. Table 6 summarizes these parameters in FAC-treated patients from Novosibirsk Municipal Hospital No 1. Further, these parameters have been correlated with survival. Patients with stage IV cancer were omitted from the analysis, as our data suggested Panagen provided little advantage to this patient group.

**Table 6:** Percent of cells on day 21 following the therapy, normalized to the initial levels before the therapy. Values above 100 indicate the cell counts have increased above the initial levels

Patient number	Breast cancer stage	CD8 + perforin + T cells		CD123+		CD11+		CD25 + CD127–	
		After the 1st round of chemotherapy	After the 3rd round of chemotherapy	After the 1st round of chemotherapy	After the 3rd round of chemotherapy	After the 1st round of chemotherapy	After the 3rd round of chemotherapy	After the 1st round of chemotherapy	After the 3rd round of chemotherapy
FAC chemotherapy + Placebo									
02–12^a^	IIIA	122.2	111.1	440.9	118.2	150.5	30.9	26.8	34.1
02–07	IIIB	46.7	105.6	80.0	176.7	43.8	200.0	220.8	54.7
FAC chemotherapy + Panagen									
02–02	IIA	300.0	420.0	92.3	615.4	92.9	333.3	1.1	81.8
02–10	IIA	178.6	178.6	543.8	37.5	666.7	83.3	120.0	62.0
02–03	IIA	100.0	23.3	61.4	59.1	41.1	41.1	4.5	116.7
02–11^a^	IIB	123.5	58.8	310.3	34.5	66.1	14.0	16.7	133.3
02–15	IIIA	33.3		26.9	23.1	54.8	19.4	69.2	69.2
02–05	IIIB	39.4	106.3	35.3	220.6	62.7	178.0	180.0	160.0
02–14	IIIB	-		153.8	123.1	97.7	85.2	127.3	118.2
02–01	IIIB	200.0	525.0	263.0	42.0	150.0	42.0	200.0	200.0
02–08	IIIB	61.8	117.6	88.2	188.2	204.8	202.4	175.0	300.0

Note: ^a^ – tamoxifen treatment. Patients who progressed or died are shown in boldface

The figures were available for 2 Placebo-treated patients with stage IIIA and IIIB disease (Table 6). The surviving patient had a pronounced trend for gradually improving adaptive immunity whereas T-reg population was significantly reduced. The other patient, who did not survive the 5-year period, had high T-reg counts in one of the tests. Adaptive immunity scores generally remained high.

In the Panagen-treated group, three patients out of four remained disease-free 5 years after the therapy. In these surviving patients, the parameters characteristic of the stimulated adaptive immunity were greatly improved. T-reg population was either slightly above the initial level or was reduced during the course of 3 chemotherapy rounds. The patient who did not survive showed no response to the treatments, as assayed by plasmacytoid and myeloid dendritic cells and activated T cell counts. This was accompanied with increasing numbers of T-regs, compared to the initial values observed in that patient.

No correlation between survival and cell counts was evident for stage III patients receiving Panagen. However, we must note that the two patients who deceased displayed 2–3 times more T-regs relatively to the initial levels, which was accompanied by an otherwise activated adaptive immunity profile. In the surviving patients, the percentage of T-regs either progressively decreased during the study or was not high at the baseline. In all but one cases, adaptive immunity appeared activated.

It is widely known that cyclophosphamide is not merely a cytotoxic drug but also has an immunomodulatory activity. It induces abortive mobilization of CD34 hematopoietic progenitors [26–28], it stimulates proliferation of dendritic cell progenitors in the bone marrow, which results in the increased dendritic cell counts in peripheral blood and is coincident with the unfolding adaptive immune response [29]. It generally increases the immune response by maintaining the balance of dendritic cell subpopulations [30], and finally it either abrogates or inhibits the functionality of T-regs [31, 32]. In this context, it is difficult to unambiguously establish the direct contribution of Panagen into changes in the parameters measured in our patients.

Taken together, our data indicate that the following combination of parameters may have a positive prognostic value: high percentage of CD8 + perforin + T-cells, high percentage of CD123+ and/or CD11+ dendritic cells and low or decreasing T-reg counts.

## Discussion

Clinical evidence indicates that upon proper therapy, stage IIIB breast cancer is treatable and shows a DFS rate of 10–40 %. DFS is below 10 % for stage IIIC. The sample size of patients in our study was relatively small, and so we grouped IIIB and IIIC stage breast cancer patients into one dataset for statistical analysis. DFS rate (17 %) of patients receiving standard of care therapy is within the global range. Patients who additionally received Panagen demonstrate a significantly higher DFS of 50 %.

Thus, the following mechanism of the anticancer activity of Panagen emerges from the above data and the pre-clinical studies published by our and other groups over the past 15 years [13, 17–21, 33–36].

In all likelihood, tablet form of Panagen acts via interaction of fragmented dsDNA with immune cells resident in the intestinal lymphoid tissue. Active substance of Panagen is delivered to the upper GI tract in the form a tablet with a gastro-resistant coating where it falls apart and the substance is dissolved in the intestinal lumen. DNA fragments eventually reach mononuclear cells of Peyer’s patches, lymphoid follicles of appendix and solitary follicles resulting in their activation [37]. Following activation, various immune cells of intestinal lymphoid tissue migrate into the lymph and blood circulation and reach immunocompetent organs. These immune cells stimulate proliferation and mobilization of hematopoietic progenitors or their immediate committed progeny via direct cell-cell contacts or cytokine secretion.

Similarly, dendritic cells resident in the intestinal lymphoid tissue become activated by dsDNA [33–36] and enter the lymph/bloodstream. Upon anchoring in lymphoid organs (mesenterium), these dendritic cells engulf cancer neo-antigens that become available as a tumor cell debris produced by the concurrent chemotherapy. These events culminate in the development of adaptive anticancer immune response.

Clearly, in order to uncover the complexity of Panagen’s anticancer activity, larger-scale clinical trials are needed. These should include massive analysis of how adaptive immunity is shaped when cytostatic drugs are given to cancer patients.

## Conclusions

Disease-free survival rate (17 %) of patients with IIIB + C stage receiving standard of care therapy is within the global range. Patients who additionally received Panagen demonstrate a significantly higher disease-free survival of 50 %. This confirms anticancer activity of Panagen.

## Abbreviations

AC chemotherapy: Chemotherapy including doxorubicin and cyclophosphamide
DFS: Disease-free survival
dsDNA: Double-stranded DNA
T-reg: CD25+ CD127– T-regulatory lymphocyte
FAC chemotherapy: Chemotherapy including 5-fluorouracil, doxorubicin and cyclophosphamide
